# Genomic analysis of Legionella pneumophila in the drinking water system of a large building over 25 years

**DOI:** 10.1099/mgen.0.001393

**Published:** 2025-05-23

**Authors:** Helena M. B. Seth-Smith, Adrian Egli, Sylvia Gautsch, Michael M. Bornstein, Eva M. Kulik

**Affiliations:** 1 Institute of Medical Microbiology, University of Zürich, Zurich, Switzerland; 2 Division of Clinical Bacteriology and Mycology, University Hospital Basel, Basel, Switzerland; 3 Applied Microbiology Research, Department of Biomedicine, University of Basel, Basel, Switzerland; 4 State Laboratory Basel-City, Basel, Switzerland; 5 Department of Oral Health & Medicine, University Center for Dental Medicine UZB, University of Basel, Basel, Switzerland; 6 Department Research, University Center for Dental Medicine UZB, University of Basel, Basel, Switzerland

**Keywords:** dental institution, evolution, monitoring, ST45, surveillance, water quality

## Abstract

*Legionella pneumophila*, the causative agent of Legionnaires’ disease, is often found in the plumbing systems of buildings, from where it can be transmitted to humans via inhalation or aspiration of contaminated water drops. Annual routine water sampling from the potable water system of an occupational healthcare building in Basel over 25 years was performed in accordance with national guidelines. Overall, 309 water samples were collected at 38 time points over the period of 25 years. *L. pneumophila* was recovered from 120 water samples (38.8%) from 26 time points. No clinical infections were recorded during this period. Initial decontamination measures were successful for ~12 years, after which an increase in the total number of *Legionella* c.f.u. as well as of *L. pneumophila*-positive sites was noticed in 2007. Whole genome sequencing (WGS) analysis of *n*=123 isolates from *n*=113 samples showed all *L. pneumophila* to be sequence type 45 (sequence-based typing scheme). The isolates are closely related, with only 408 SNPs among all isolates after the bioinformatic removal of recombination events. Over the 25 years, a single lineage deriving from a recent common ancestor colonized the water system of this building. The phylogeny of isolate genomes can be interpreted as inferring good water circulation and possible recolonization from a common source after cleaning, with genome evolution and insertion/loss of large elements evident. Regular monitoring of waterlines in healthcare settings helps to identify concentrations of *Legionella* spp., and WGS is recommended for detailed investigation.

**Impact Statement**

This is the most detailed, long-term study of *Legionella pneumophila* in the water system of a single building recorded to date. The *L. pneumophila* isolates found in the building over the sampling period of 25 years were all closely related, belonging to ST45. SNP analysis suggested that the common ancestor of the cluster was from around 1938 (range 1911–1959), and movement of a large genomic island and plasmid transfer were observed. Despite several decontamination measures, it was impossible to completely eradicate *Legionella* spp. from the water system of the historic building. No infections could be attributed to the presence of *L. pneumophila* in this building. To mitigate the risk of legionellosis from such buildings, awareness, regular water testing based on official national guidelines and recommendations and other control measures, such as the use of sterile water for critical procedures, can be recommended.

## Data Summary

All data are submitted to the ENA under project PRJEB79004 under accession nos. ERR13662450–ERR13662572 (Table S1). Supplementary material is available on Figshare: https://doi.org/10.6084/m9.figshare.28387712.v1 [1].

## Introduction

*Legionella pneumophila*, a Gram-negative, aerobic and non-spore-forming bacterium, is an opportunistic pathogen that can present a risk to human health. It can cause Legionnaires’ disease, a severe atypical pneumonia, or Pontiac fever, a milder form of the same infection [2]. Of the over 60 known *Legionella* species, *L. pneumophila* serogroup 1 is responsible for most severe infections. Transmission from environment to human occurs through inhalation or aspiration of aerosols containing *L. pneumophila*, where the bacteria infect alveolar macrophages in the lung [3,4].

*L. pneumophila* are ubiquitous in various freshwater habitats as well as man-made water systems such as potable water systems, fountains, spa water and air-conditioner cooling towers [2,5]. In these environments, *L. pneumophila* can be found planktonically or coexisting with other micro-organisms in biofilms where they can parasitize and replicate within free-living amoebae [6]. The association with amoebae offers benefits to *Legionella*, providing protection against disinfectants, UV radiation or fluctuations in water temperature [7]. Water temperatures between 25 and 45 °C are optimum for growth, at which *L. pneumophila* may reach critical concentrations. At higher water temperatures, bacterial growth is inhibited, and temperatures above 60 °C start to be bactericidal for *L. pneumophila*.

Due to this ability to grow within pipes, guidelines and regulations exist to control the growth of *Legionella* spp. in the water systems of buildings. In addition to sanitary measures such as regular maintenance of the waterlines or temperature checks, regular microbiological analysis of water samples is recommended to monitor the water quality [8,9]. This also applies to healthcare facilities, such as hospitals or clinics, which may treat vulnerable persons having a potential higher risk of acquiring Legionnaire’s disease. In dental institutions, biofilm formation in dental unit water lines may result in high numbers of micro-organisms including *Legionella* spp. in the water used for cooling or ultrasonication procedures [10]. Consequently, an increased risk for *Legionella* infection has also been assumed for dental health care workers; however, a recent review suggests that there is only very limited evidence for an increased risk in this professional group [11].

Epidemiological studies based on the analysis of genomic data are very helpful in identifying transmission routes and tracing potential sources of infections. In addition, such studies can also be useful to assess the microbial diversity and evolutionary relatedness in various environments. Genomic epidemiological analysis, together with aggressive interventional measures, was central to controlling an outbreak of nosocomial cases of *L. pneumonia* in an Australian hospital [12]. In this case, eradication methods, which included disinfection of the water distribution system with a chlorinated, alkaline detergent as well as removal of redundant plumbing systems, appeared to be successful in eliminating the *L. pneumophila* population responsible for the infections in the hospital for at least six months. However, other studies show that artificial water systems remain colonized by *L. pneumophila* despite the implementation of decontamination measures such as the superheat and flush method [13,14].

At the Dental School of the University of Basel, Switzerland, routine water testing began in the early 1990s with a focus on tap water to assess and control bacterial contamination at the source level before it could enter the waterlines of dental units. In this context, a unique collection of *L. pneumophila* strains isolated from different points in the water system was created, before and after decontamination measures, in the same water distribution system, from 1994 to 2018. In summer 2019, the Dental School of the University of Basel moved to a new building in a different location.

The aim of this study was to investigate the distribution and evolution of *L. pneumophila* within a single building over 25 years.

## Methods

### Study setting and sample collection

Routine testing of the drinking water system was performed annually at the Dental School of the University of Basel, Switzerland, on a regular basis. The building has housed the Dental School of the University of Basel for almost one hundred years and was, where necessary, modified to meet new requirements during this period. In particular, the historic part of the building contained older water pipes and was not initially designed to prevent potential colonization by *Legionella* spp.

Therefore, the sampling points were selected based on a risk assessment and included end points of the water distribution system as well as rarely used faucets. Additional tests were carried out as required and after consultation with the Hygiene Commission of the building. Water samples were usually taken from the warm water circuit and processed in accordance with national guidelines that were in force at the respective time [9]. Following species identification, *L. pneumophila* isolates were stored at −70 °C until analysis. As required by the guidelines, 200 ml of water was taken from the respective water outlet and filtered by pressure through a membrane filter. The filter was then placed onto an agar plate selective for *Legionella* species. Prior to 2008, *Legionella* MWY selective medium (Oxoid, Pratteln, Switzerland) was used, whereas from 2008 on, GVPC medium (Oxoid, Thermo Scientific, Reinach, Switzerland) was used for the initial isolation of *Legionella* spp. from the water samples. When reporting the total c.f.u., numbers are calculated to 1 l of water.

### Culture and DNA extraction

*L. pneumophila* strains isolated during the routine water tests in the years 1994 to 2018, i.e. during a period of 25 years, were included in this study. The isolates were grown on buffered charcoal yeast extract agar (Thermo Fisher Scientific, Reinach, Switzerland) aerobically in a humidified atmosphere at 37 °C for 48–72 h. DNA was extracted using the Qiagen EZ1 with the DNA tissue kit (Qiagen AG, Hombrechtikon, Switzerland).

### Genome sequencing and analysis

All samples were sequenced on the NextSeq500 PE150 after Illumina DNA prep, resulting in over 30× mean read depth per sample. Isolate ZIB8567 was sequenced by Oxford Nanopore Technologies on an R10.4 flowcell to a mean read depth of 50× and base called with Dorado hac@v4.2.0 (https://github.com/nanoporetech/dorado). All data were submitted to the European Nucleotide Archive (ENA) under project PRJEB79004.

Ridom v 9.0.10 was used on Unicycler v0.3.0b [15] assemblies to assign sequence-based typing sequence types (STs) to the isolates. Data on two ST45 isolates (917,837 and 919,569) were obtained from the UK Health Security Agency (UKHSA) and used as outgroups.

For phylogenetic analysis, the hybrid Unicycler v0.4.8 [15] assembled genome of isolate ZIB8567 (isolated in 2018), comprising a single contig of 3,522,222 bp (accession OZ182546.1), was used as the reference against which to map the reads of all isolates within CLC Genomics Workbench v22.0.2, calling variants with parameters: variant calling with 10× minimum coverage, 10 minimum count and 70% minimum frequency. Consensus genomes were extracted from mapped reads using a minimum coverage of 10. A multiple sequence alignment was created from the whole genome alignments, exported and run through Gubbins v3.3.0 [16] with five iterations to remove recombinations. Results were viewed in Phandango [17]. BactDating v1.1.1 [18] was run four times on the Gubbins output with one million iterations and the arc model to check the reproducibility, and representative results are given. Abricate v1.0.1 (https://github.com/tseemann/abricate) and the NCBI database [19] were used to define resistance determinants present.

Regions of the genome present in the sequenced genomes and absent from the genomes of strain Philadelphia (SRR801743 and ERR351242), or present at very low coverage, were identified by analysing the mapping statistics of all isolates in CLC (<99.9% of reference mapped to), visualizing mapped reads in Artemis and marking regions without mapping. Regions over 1 kb were compared with blastn against the nt database to check whether there were matches in other submitted genomes, and top matches are given. Insertion elements (ISs) were categorized using ISFinder (https://isfinder.biotoul.fr). Further elements were characterized by iterative analysis of the mapping statistics (<98% reads mapped) and *de novo* assembly of Illumina data in CLC using default parameters (slow) and mapping of reads as above. Loss and gain of elements were confirmed using Panaroo v1.3.0 [20] on assemblies created with Unicycler v0.4.8 [15] annotated with Bakta v1.9.3 [21] within the IMMense pipeline (https://gitlab.uzh.ch/appliedmicrobiologyresearch/immense), and assessing resulting gene presence/absence files in Phandango [17].

## Results

### Sampling

**Fig. 1.**
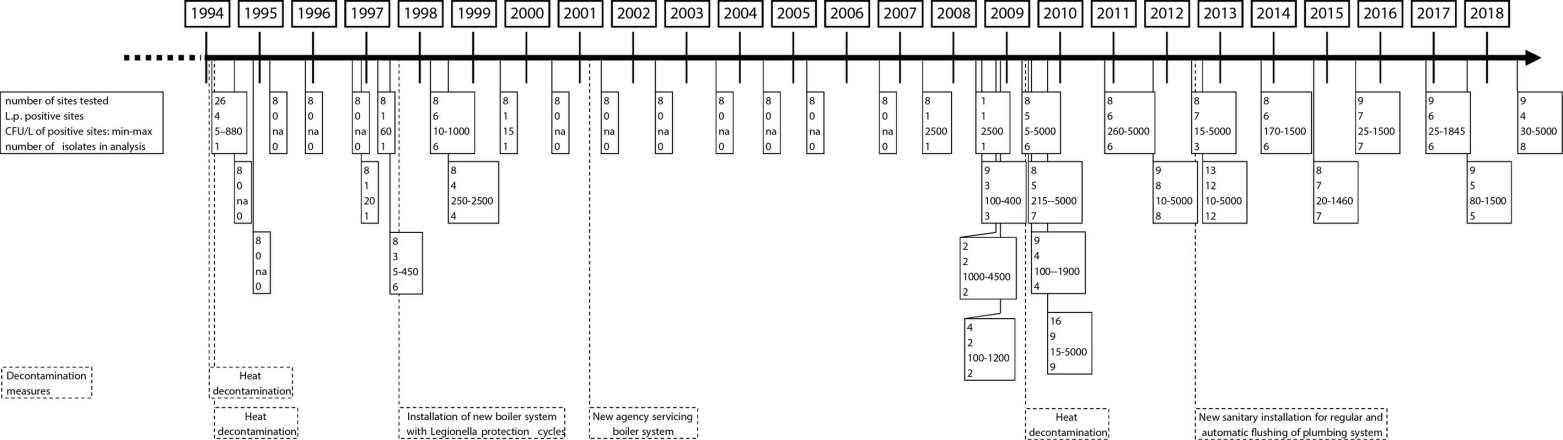
Timeline of *L. pneumophila* strains isolated over 25 years. Shown are the number of sites tested per time point, the number of sites that were *L. pneumophila* positive, the minimum and maximum c.f.u. l^−1^ values for *L. pneumophila*-positive sites per time point and the number of isolates per time point that was analysed (in bold) as well as the decontamination measures carried out during this time period.

Overall, 309 water samples, taken from different locations in the building, were collected at 38 time points over a period of 25 years. As the building, including waterlines, was renovated over the course of the 25 years at various locations, the same sampling points might not have been accessible any longer, and therefore, nearby faucets fed by the same waterline were selected. *L. pneumophila* was recovered from 120 water samples (38.8%) and 113 *L*. *pneumophila* isolates from 26 time points could be included in this study (Fig. 1), as well as 10 replicate isolates picked from the same plates as 5 of the isolates. After initial control measures, which to this date consisted mainly of two cycles of heat decontamination and the installation of a new boiler system, which were successful for ~11 years until 2007, an increase in the total number of *Legionella* c.f.u. as well as of *L. pneumophila*-positive sites was noticed (Fig. 1).

### Phylogenetic analysis of ST45 cluster

All 123 isolates from the building belong to ST45, which is within serogroup 1. They are unrelated to previously published samples from other locations within the City of Basel, where the Dental School of the University of Basel is located [22].

**Fig. 2.**
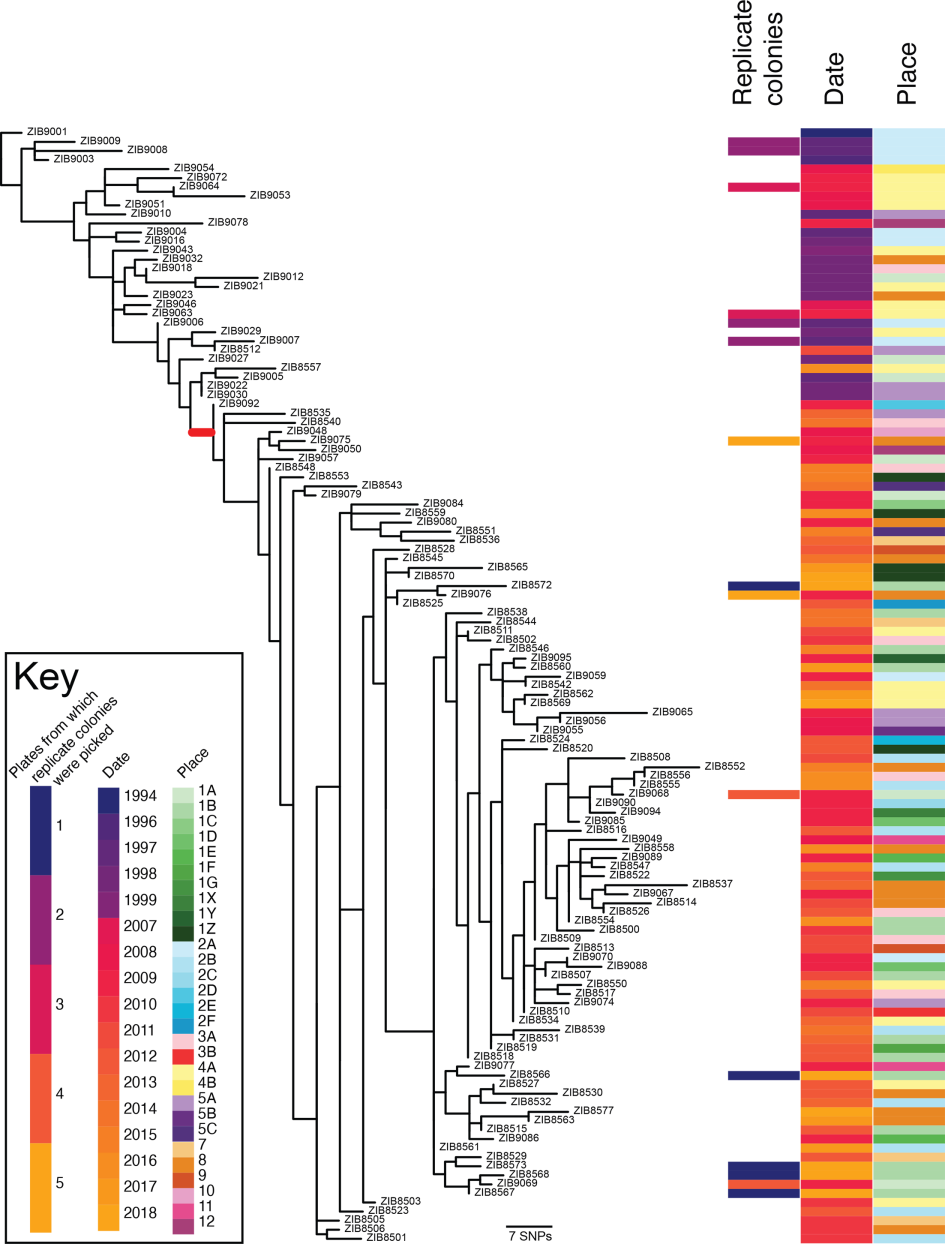
Recombination-adjusted phylogenetic tree of all isolates with associated metadata. Gubbins generated a phylogeny based on mapping all data against a complete hybrid assembly of ZIB8567, rooted on the oldest isolate (ZIB9001, 1994). Metadata (right) shows (left to right) which replicate isolates came from the same plates (replicate colonies), year of isolation (date) and location of isolation within the building (place). As some of the same sampling points were not always accessible during the 25 years, nearby faucets fed by the same waterline were selected and/or additional water outlets were included. Water outlets fed by the same waterline are represented by the same number. Figure generated using Phandango [17]. The branch shown in red carries samples isolated exclusively after 2008.

Phylogenetic analysis of the 123 isolates, based on a complete genome hybrid assembly reference from an isolate within the cluster, shows that they are closely related (Fig. 2). We found 408 SNPs across the whole cluster, with recombinations removed. There is no apparent clustering of isolates taken from the same sampling points. Similarly, there is no clear clustering between replicate colonies which were taken from some agar plates in 1997, 2009 and 2018, with samples from the same plates being found at diverse locations in the tree (Fig. 2). The branch which particularly expanded post-2008 (Fig. 2) carries a C-A SNP at position 377 of the transfer-messenger RNA (tm-RNA) *ssrA*.

A mutation rate of 0.38 SNPs per genome per year (range 0.27–0.49) and a most likely date for the common ancestor of this cluster of 1938 (range 1911–1959) were calculated using BactDating. The two ST45 isolates submitted to the UKHSA originating from Oxford in 2009 (917,837) and of unknown provenance (919,569) are, respectively, 1,875 and 1,888 SNPs from the ST45 isolates sequenced for this study (recombinations removed). The recombinations identified within the cluster represent 94% of the identified SNPs (6,876/7,284), although many fall within repeat genes and may represent mapping difficulties (Fig. S1, available in the online Supplementary Material).

Regarding resistance determinants, the gene aph(9)-Ia, a chromosomally encoded aminoglycoside phosphotransferase, was identified in the hybrid-assembled genome of *L. pneumophila* ZIB8567 with a nucleotide identity of 89.81%.

### Regions of difference and genomic islands

All the presented *L. pneumophila* genomes share very similar gene content, with reads from all genomes mapping to over 99% of the ZIB8567 hybrid assembly. This is also the case for the ST45 genomes 917,837 and 919,569 obtained from UKHSA. Reads from the *L. pneumophila* strain Philadelphia, however, cover only 87% of the assembly, with under 90% of the reads mapping.

Many regions of difference (RoD) were identified in the ST45 genomes which are not present in the Philadelphia reference strain genome (Table 1). When comparing the two ST45 database genomes, only one possible genomic island (GI) appears to be unique to the isolates from this study: a 30 kb region (172,917–203,527 in the ZIB8567 hybrid assembly) adjacent to the tm-RNA *ssrA*, sharing 90.55% identity over 92% of its length with a region of the genome of *Legionella longbeachae* strain B1445CHC (CP045306.1). This RoD shows a higher read depth than the rest of the genome, suggesting that it exists in multi-copy and carries phage-like genes with a type IV secretion system. This RoD is absent from the genomes of six isolates, namely ZIB8547, ZIB9003, ZIB9004, ZIB9008, ZIB9009 and ZIB9016, all originating from close sampling points 2A and 2B. The position of these isolates in the phylogeny infers that the ancestral strain of this cluster carried the RoD, and it had been lost on three independent occasions within the phylogeny.

**Table 1.** List of RoDs identified in the isolates. Locations refer to the hybrid assembly of isolate ZIB8567, accession no. OZ182546.1

Start	End	Length	Found in	blastn query cover	blastn percent identity	Match to strain accession	Comment
57,502	60,351	2,850	All ST45 genomes	100.00	99.79	Lorraine FQ958210.1	Homology to ISLpn9
65,009	66,795	1,787	All ST45 genomes	100.00	100.00	Lorraine FQ958210.1	
69,573	75,226	5,654	All ST45 genomes	100.00	100.00	Lorraine FQ958210.1	
107,571	108,853	1,283	All ST45 genomes	100.00	100.00	Lorraine FQ958210.1	
154,181	155,180	1,000	All ST45 genomes	100.00	99.60	Lorraine FQ958210.1	Homology to ISLpn9
172,917	184,420	11,504	Unique to ZIB genomes	80.00	89.22	*L. longbeachae* strain B1445CHC CP045306.1	Present at much higher coverage
197,488	203,527	6,040	Unique to ZIB genomes	100.00	98.24	*L. longbeachae* strain B1445CHC CP045306.1	Present at much higher coverage
203,528	228,760	25,233	All ST45 genomes	100.00	100.00	Lorraine FQ958210.1	
254,469	255,506	1,038	All ST45 genomes	100.00	100.00	Lorraine FQ958210.1	
258,026	259,597	1,572	All ST45 genomes	100.00	100.00	Lorraine FQ958210.1	
261,477	263,700	2,224	All ST45 genomes	100.00	99.69	Lorraine FQ958210.1	Homology to ISLpn9
310,472	311,473	1,002	Very low coverage in Philadelphia reference	100.00	99.80	Lorraine FQ958210.1	Homology to ISLpn9
333,355	336,147	2,793	All ST45 genomes	100.00	99.96	Lorraine Q958210.1	
359,496	360,497	1,002	All ST45 genomes	100.00	99.90	Lorraine FQ958210.1	Homology to ISLpn9
364,046	366,198	2,153	All ST45 genomes	100.00	100.00	Lorraine FQ958210.1	
388,083	390,250	2,168	All ST45 genomes	100.00	100.00	Lorraine FQ958210.1	
392,528	393,528	1,001	All ST45 genomes	100.00	99.80	Lorraine FQ958210.1	Homology to ISLpn9
470,948	471,949	1,002	Very low coverage in Philadelphia reference	100.00	99.90	Lorraine FQ958210.1	Homology to ISLpn9
492,613	493,614	1,002	All ST45 genomes	100.00	99.80	Lorraine FQ958210.1	Homology to ISLpn9
523,278	524,733	1,456	Very low coverage in Philadelphia reference	100.00	99.93	Lorraine Q958210.1	Homology to ISLpn9
578,328	579,663	1,336	All ST45 genomes	100.00	100.00	NCTC11985 LT906452.1	
620,762	622,020	1,259	All ST45 genomes	100.00	100.00	NCTC11985 LT906452.1	
622,674	660,984	38,311	All ST45 genomes	75.00	98.01	Corby CP000675.2	
698,965	699,965	1,001	All ST45 genomes	100.00	99.80	NCTC11985 LT906452.1	Homology to ISLpn9
743,322	746,237	2,916	All ST45 genomes	100.00	100.00	NCTC11985 LT906452.1	
782,582	791,077	8,496	All ST45 genomes	100.00	99.80	NCTC11985 LT906452.1	Across toxin gene *rtxA*
795,792	798,713	2,922	All ST45 genomes	100.00	100.00	NCTC11985 LT906452.1	
812,799	818,434	5,636	All ST45 genomes	100.00	100.00	NCTC11985 LT906452.1	
925,770	927,658	1,889	All ST45 genomes	99.00	97.78	ERS1305867 CP048618.1	
944,897	946,813	1,917	All ST45 genomes	100.00	98.44	Lorraine FQ958210.1	
1,048,512	1,050,216	1,705	All ST45 genomes	100.00	97.65	ERS1305867 CP048618.1	
1,059,149	1,060,776	1,628	Very low coverage in Philadelphia reference	100.00	99.63	PATHC002 CP113439.1	Homology to ISLpn9
1,165,326	1,166,952	1,627	Very low coverage in Philadelphia reference	100.00	99.82	Lorraine FQ958210.1	Homology to ISLpn9
1,181,211	1,182,982	1,772	All ST45 genomes	100.00	100.00	Lorraine FQ958210.1	
1,193,911	1,195,503	1,593	All ST45 genomes	100.00	100.00	Lorraine FQ958210.1	
1,202,863	1,205,380	2,518	All ST45 genomes	100.00	99.80	Lorraine FQ958210.1	Homology to ISLpn9
1,227,451	1,231,629	4,179	All ST45 genomes	100.00	99.88	Lorraine FQ958210.1	
1,233,358	1,235,843	2,486	All ST45 genomes	100.00	100.00	Lorraine FQ958210.1	
1,237,708	1,239,143	1,436	All ST45 genomes	100.00	100.00	Lorraine FQ958210.1	
1,333,023	1,337,543	4,521	All ST45 genomes	100.00	99.93	Lorraine FQ958210.1	
1,355,443	1,356,680	1,238	All ST45 genomes	100.00	100.00	Lorraine FQ958210.1	
1,358,599	1,360,228	1,630	Very low coverage in Philadelphia reference	100.00	99.94	Lorraine FQ958210.1	Homology to ISLpn9
1,439,798	1,441,093	1,296	Very low coverage in Philadelphia reference	100.00	99.46	PATHC002 CP113439.1	Homology to ISLpn9
1,477,327	1,479,726	2,400	All ST45 genomes	100.00	99.62	PATHC002 CP113439	Homology to ISLpn9
1,593,111	1,594,428	1,318	Very low coverage in Philadelphia reference	100.00	99.85	Lorraine FQ958210.1	Homology to ISLpn9
1,595,609	1,597,261	1,653	All ST45 genomes	100.00	100.00	Lorraine FQ958210.1	Lower homology to ISLpn9
1,605,942	1,606,940	999	All ST45 genomes	100.00	100.00	Lorraine FQ958210.1	Homology to ISLpn9
1,627,254	1,629,188	1,935	All ST45 genomes	100.00	100.00	NCTC11985 LT906452.1	
1,671,268	1,677,430	6,163	All ST45 genomes	100.00	100.00	Lorraine FQ958210.1	
1,729,636	1,732,750	3,115	All ST45 genomes	100.00	100.00	Lorraine FQ958210.1	
1,740,199	1,741,299	1,101	All ST45 genomes	100.00	99.64	NCTC11985 LT906452.1	Homology to ISLpn9
1,750,367	1,752,008	1,642	Very low coverage in Philadelphia reference	100.00	99.70	Lorraine FQ958210.1	Homology to ISLpn9
1,778,688	1,781,029	2,342	All ST45 genomes	100.00	100.00	Lorraine FQ958210.1	
1,869,245	1,871,275	2,031	All ST45 genomes	100.00	100.00	Lorraine FQ958210.1	
1,896,804	1,900,876	4,073	Very low coverage in Philadelphia reference	100.00	99.80	Lorraine FQ958210.1	Homology to ISLpn9
1,971,818	1,972,865	1,048	Very low coverage in Philadelphia reference	100.00	99.90	Lorraine FQ958210.1	Homology to ISLpn9
2,017,691	2,018,953	1,263	All ST45 genomes	100.00	100.00	Lorraine FQ958210.1	Homology to ISLdr1
2,061,021	2,062,670	1,650	All ST45 genomes	100.00	100.00	Lorraine FQ958210.1	
2,067,226	2,070,972	3,747	Very low coverage in Philadelphia reference	100.00	100.00	Lorraine FQ958210.1	Homology to ISLpn9
2,080,183	2,081,962	1,780	All ST45 genomes	100.00	100.00	Lorraine FQ958210.1	
2,100,255	2,102,480	2,226	Very low coverage in Philadelphia reference	100.00	99.91	Lorraine FQ958210.1	Homology to ISLpn9
2,127,224	2,133,200	5,977	All ST45 genomes	100.00	100.00	Lorraine FQ958210.1	
2,147,413	2,150,629	3,217	All ST45 genomes	100.00	100.00	Lorraine FQ958210.1	
2,152,162	2,153,520	1,359	All ST45 genomes	100.00	100.00	Lorraine FQ958210.1	
2,178,245	2,180,163	1,919	Very low coverage in Philadelphia reference	100.00	99.79	Lorraine FQ958210.1	Homology to ISLpn9
2,190,825	2,197,681	6,857	All ST45 genomes	100.00	99.97	PATHC002 CP113439.1	
2,216,894	2,217,895	1,002	Very low coverage in Philadelphia reference	100.00	99.90	PATHC002 CP113439.1	Homology to ISLpn9
2,266,139	2,267,687	1,549	All ST45 genomes	100.00	100.00	Lorraine FQ958210.1	
2,286,060	2,289,391	3,332	All ST45 genomes	100.00	100.00	Lorraine FQ958210.1	
2,292,710	2,314,659	21,950	All ST45 genomes	100.00	100.00	Lorraine FQ958210.1	
2,315,009	2,316,667	1,659	Very low coverage in Philadelphia reference	100.00	100.00	Lorraine FQ958210.1	
2,319,117	2,324,373	5,257	All ST45 genomes	100.00	1005.00		
2,327,309	2,331,362	4,054	All ST45 genomes	100.00	100.00	Lorraine FQ958210.1	
2,367,178	2,368,459	1,282	All ST45 genomes	100.00	100.00	Lorraine FQ958210.1	
2,374,126	2,376,841	2,716	All ST45 genomes	100.00	100.00	Lorraine FQ958210.1	
2,395,108	2,396,866	1,759	All ST45 genomes	100.00	100.00	Lorraine FQ958210.1	
2,398,121	2,399,129	1,009	Very low coverage in Philadelphia reference	100.00	99.70	Lorraine FQ958210.1	Homology to ISLpn9
2,437,537	2,438,802	1,266	All ST45 genomes	100.00	100.00	Lorraine FQ958210.1	
2,530,688	2,531,689	1,002	Very low coverage in Philadelphia reference	100.00	99.80	Lorraine FQ958210.1	Homology to ISLpn9
2,562,295	2,563,296	1,002	All ST45 genomes	100.00	100.00	NCTC11985 LT906452.1	Homology to ISLpn9
2,624,142	2,697,268	73,127	All ST45 genomes	100.00	99.99	Lorraine FQ958210.1	
2,700,063	2,708,857	8,795	All ST45 genomes	100.00	99.73	Lorraine FQ958210.1	
2,711,396	2,713,990	2,595	All ST45 genomes	100.00	100.00	Lorraine FQ958210.1	
2,720,705	2,728,751	8,047	All ST45 genomes	100.00	100.00	Lorraine FQ958210.1	
2,755,536	2,756,800	1,265	All ST45 genomes	100.00	100.00	Lorraine FQ958210.1	
2,760,534	2,762,270	1,737	All ST45 genomes	100.00	100.00	Lorraine FQ958210.1	
2,774,564	2,776,814	2,251	All ST45 genomes	100.00	100.00	Lorraine FQ958210.1	
2,784,580	2,788,461	3,882	All ST45 genomes	100.00	100.00	Lorraine FQ958210.1	
2,871,046	2,873,896	2,851	Very low coverage in Philadelphia reference	100.00	100.00	PATHC002 CP113439.1	Homology to ISLpn9
2,877,959	2,880,331	2,373	All ST45 genomes	100.00	99.96	Lorraine FQ958210.1	
2,901,276	2,903,059	1,784	Very low coverage in Philadelphia reference	100.00	99.94	Lorraine FQ958210.1	Homology to ISLpn9
2,927,856	2,930,694	2,839	All ST45 genomes	100.00	100.00	Lorraine FQ958210.1	
2,938,791	2,941,692	2,902	All ST45 genomes	100.00	99.97	Lorraine FQ958210.1	Homology to ISLpn11
2,944,637	2,949,035	4,399	All ST45 genomes	100.00	100.00	Lorraine FQ958210.1	
2,949,872	2,951,925	2,054	All ST45 genomes	100.00	100.00	Lorraine FQ958210.1	
2,967,128	2,974,009	6,882	All ST45 genomes	100.00	100.00	Lorraine FQ958210.1	
2,980,896	2,986,006	5,111	All ST45 genomes	100.00	100.00	Lorraine FQ958210.1	
2,988,838	2,993,936	5,099	All ST45 genomes	100.00	100.00	Lorraine FQ958210.1	
2,996,660	3,000,260	3,601	All ST45 genomes	100.00	100.00	Lorraine FQ958210.1	
3,002,511	3,004,154	1,644	All ST45 genomes	100.00	100.00	Lorraine FQ958210.1	
3,022,889	3,025,113	2,225	All ST45 genomes	100.00	100.00	PATHC002 CP113439.1	Homology to ISLpn9
3,135,943	3,137,185	1,243	All ST45 genomes	100.00	100.00	Lorraine FQ958210.1	
3,210,927	3,212,090	1,164	Very low coverage in Philadelphia reference	100.00	98.63	Lorraine FQ958210.1	Homology to ISLpn9
3,322,005	3,323,006	1,002	Very low coverage in Philadelphia reference	100.00	99.90	Lorraine FQ958210.1	Homology to ISLpn9
3,380,474	3,381,474	1,001	Very low coverage in Philadelphia reference	100.00	99.70	Lorraine FQ958210.1	Homology to ISLpn9
3,395,571	3,398,446	2,876	All ST45 genomes	100.00	100.00	Lorraine FQ958210.1	
3,405,473	3,406,634	1,162	All ST45 genomes	100.00	100.00	Lorraine FQ958210.1	
3,411,700	3,413,038	1,339	All ST45 genomes	100.00	100.00	Lorraine FQ958210.1	
3,413,490	3,416,746	3,257	All ST45 genomes	100.00	100.00	Lorraine FQ958210.1	
3,462,908	3,464,229	1,322	All ST45 genomes	100.00	99.62	Lorraine FQ958210.1	Homology to ISLpn9
3,494,277	3,495,904	1,628	Very low coverage in Philadelphia reference	100.00	99.63	PATHC002 CP113439.1	Homology to ISLpn9
3,520,489	3,521,921	1,433	All ST45 genomes	94.00	99.93	H3 P114576.1	

All the rest of the RoD between ST45 and Philadelphia are present in the genomes of other strains of *L. pneumophila*, with most matches to that of strain Lorraine (FQ958210.1). Forty of the RoDs appear to be IS elements, of which the vast majority (*n*=38) are related to ISLpn9 (Table 1).

The isolates ZIB8557, ZIB9043, ZIB9046, ZIB9051, ZIB9053, ZIB9054, ZIB9063, ZIB9064 and ZIB9072, all from locations 4A and 4B, carry a 150 kb plasmid identical to pB3526CGC_150 k from *L. longbeachae* (CP042253.1). This plasmid carries *bla*OXA-29, among other potential antimicrobial resistance encoding genes and those potentially encoding conjugation apparatus (Files S1 and S2). According to the phylogeny, this suggests acquisition of the plasmid on three separate occasions.

## Discussion

*L. pneumophila* has been reported from the water systems of large buildings and hospitals in studies from Canada [23], the USA [13,24, 25], Italy [26], Germany [27], Finland [14], Poland [28], Scotland [29] and Australia [12]. Genome sequencing has previously been used in such studies [12,30, 31], but none over such a long period of time as in our study.

Our data show a long-term presence of *L. pneumophila* ST45 within an old building in Basel in which the Dental School of the University of Basel was located from 1924 to 2019. The estimated time of the most recent common ancestor of the cluster, at around 1938, would therefore fit with the age and development of the building, as this historical building has been regularly renovated and new annexes added [32]. While some buildings have been found to contain multiple STs [31], our study describes the same ST found in all locations at all time points, suggesting continuous colonization of the waterlines with the same clonal strain over 25 years. That there was no clear phylogenetic signal related to location within the building infers circulation through the waterlines and less spatial structuring than described by David *et al*. [31]. Likewise, no influence of the decontamination measures could be noted in the phylogeny in terms of the extinction of certain branches. The increase in prevalence observed from 2007 could partly reflect a change in the Standard Operating Protocol (SOP) to use a different selective agar medium, although isolates indeed grew on the first medium from 1994 to 2007. Because of this increase, heat decontamination was carried out in 2009, but this did not lead to a reduction in the culture of *Legionella* spp. One SNP of interest is that in *ssrA*, which is also adjacent to a possible mobile GI; inhibition of expression of this tm-RNA [33] has severely deleterious effects on bacterial growth [34]. It is possible that a more in-depth analysis including more isolates from each time point might have resulted in a more nuanced picture in terms of clonal extinction, as decontamination measures have been shown to reduce the number of *Legionella* present [35,36]. This might also have helped us to capture the presence of any *L. longbeachae* isolates.

Investigation of global data provided by UKHSA and from the literature shows that *L. pneumophila* ST45 has previously been isolated in environmental samples from China, Japan, South Korea, France, Germany, Belgium and Italy, and in clinical isolates (*n*=41) from the USA, Canada and across Europe, including from two locations in Switzerland and the neighbouring state in Germany (Baden-Württemberg) between 2010 and 2013 [37,42]. Very few genomes are available for comparison, and the two ST45 genomes available were over 1,800 SNPs away from the presented cluster. Isolates within this ST have been found to cause clinical disease, yet no infections were identified within our study. It is commonly assumed that dental personnel have an elevated occupational risk for *Legionella* infection, as they might potentially inhale contaminated aerosols generated by water-cooled rotary instruments used in dentistry [43]. However, no cases of *Legionella* infection were reported over the course of our study period among dental personnel with direct patient contact working at the Dental School of the University of Basel (data not shown). This is in accordance with a recent meta-analysis that concluded that the occupational risk for an occupationally acquired *Legionella* infection for dental personnel is lower than previously anticipated [11] and strict compliance with currently valid guidelines may have contributed to the absence of *Legionella* infections among dental personnel at the Dental School of the University of Basel. Nevertheless, awareness, regular testing and other control measures, e.g. the use of sterile water for critical procedures, are recommended.

The calculated mutation rate within this cohort of 0.38 SNPs per genome per year compares well to previous estimates of 0.71 SNPs per genome per year (44) and 0.49 SNPs per genome per year (45). *L. pneumophila* is known to be a recombinogenic species [46,47]; the present study shows limited recombination, as is likely in an environment with limited genomic diversity, analysis of which may be confounded by repetitive genes. Recombinations around *hemA*, identified as a hotspot in ST1 [47], were not identified in this ST45 cluster. Plasmid movement and GI loss were also observed, showing that genome plasticity is possible in this population. Interestingly, these events are associated with isolates from particular locations; however, it is not possible to infer specific selective pressure on these elements within these niches. Genetic elements of * L. longbeachae* could be detected, although this bacterium itself was not isolated in this study. Other *Legionella* species, such as *Legionella geestiana* and *Legionella anisa*, were detected, albeit rarely (data not shown).

In conclusion, the present work demonstrates that a single clonal lineage of *L. pneumophila* can, despite decontamination measures, persist in a building’s water system for over 25 years. The phylogeny of the isolates can be interpreted as inferring good water circulation and possible recolonization from a common source after cleaning.

## Supplementary material

10.1099/mgen.0.001393 Uncited Table S1.

10.1099/mgen.0.001393 Uncited Fig. S1.

## Abbreviations

CFU: colony forming units
GI: genomic island
IS: insertion sequence
RoD: regions of difference
SNP: single nucleotide polymorphism
SOP: standard operating procedure
ST: sequence type
tm-RNA: transfer-messenger RNA
UKHSA: UK health security agency
WGS: whole genome sequencing
