# Supplementary material for: Genomic analysis of Legionella pneumophila in the drinking water system of a large building over 25 years
Source: Microb Genom. 2025 May 23;11(5):001393. doi: 10.1099/mgen.0.001393 (PMC12282300; doi:10.1099/mgen.0.001393)
Supplement: Uncited Fig. S1. [file mgen-11-01393-s002.pdf]

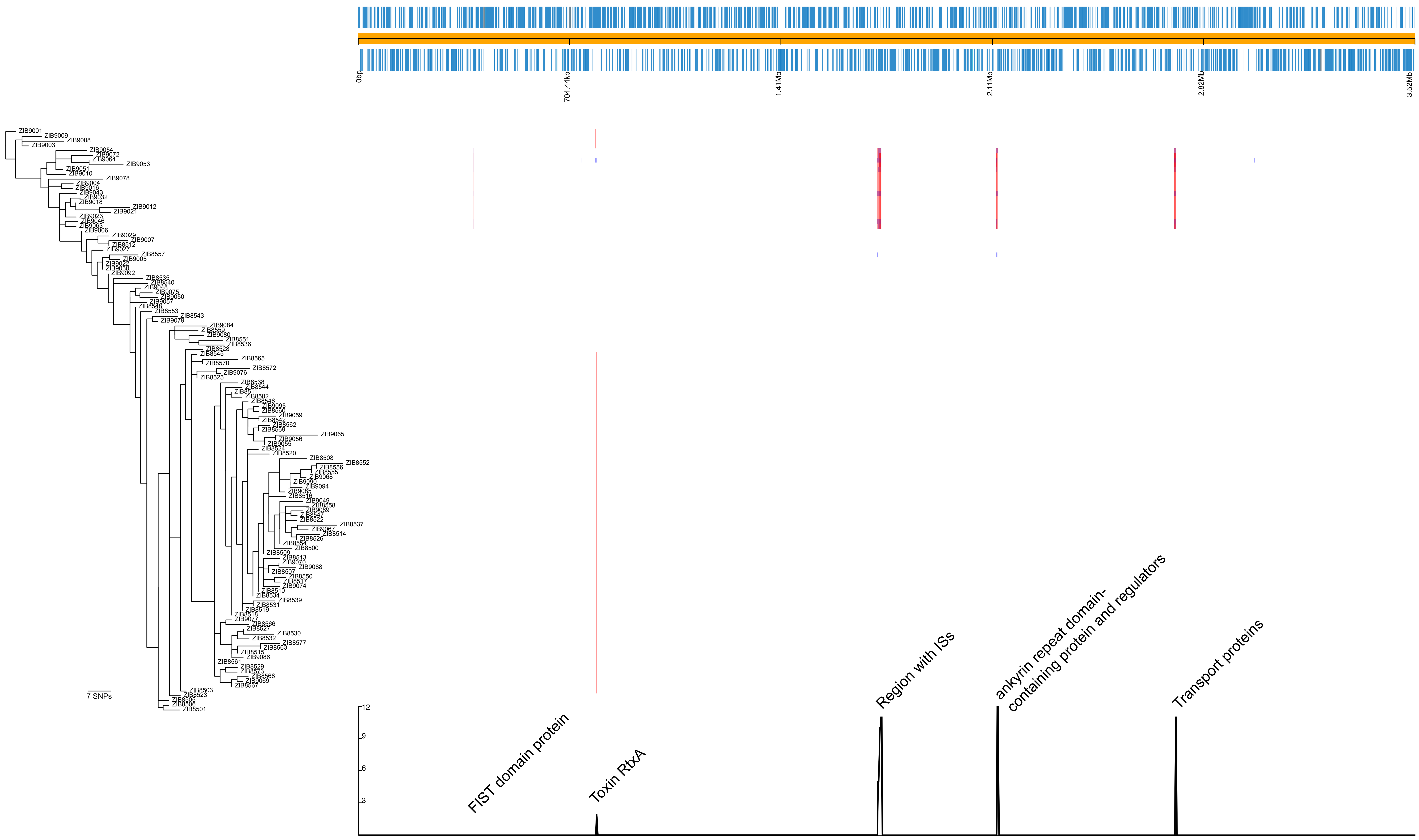

Figure S1. Recombinations identified within the ZIB cluster. Gubbins identified recombinations are shown aligned with the phylogeny rooted as in Figure 2. Genome annotation above the recombinations shows the coding sequences (CDSs) in blue along the chromosome. Below the recombination tracks, where recombination loci in single isolates are shown in blue, and those identified in more than one isolate are shown in red, is a graph of frequency with key locations annotated. Figure generated using Phandango (15).
